# Human Papillomavirus (HPV) infection in pregnant women and mother-to-child transmission of genital HPV genotypes: a prospective study in Spain

**DOI:** 10.1186/1471-2334-9-74

**Published:** 2009-05-27

**Authors:** Xavier Castellsagué, Teresa Drudis, Maria Paz Cañadas, Anna Goncé, Ramón Ros, José M Pérez, M Jesús Quintana, Jesús Muñoz, Ginesa Albero, Silvia de Sanjosé, F Xavier Bosch

**Affiliations:** 1 Unit of Infections and Cancer (UNIC), Cancer Epidemiology Research Program (CERP), IDIBELL – Institut Català d'Oncologia (ICO), Hospitalet de Llobregat, Barcelona, Spain; 2 Department of Pathology, European Institute of Oncology, Milan, Italy; 3 Molecular Biology, General Lab, Barcelona, Spain; 4 Institut Clínic de Ginecologia Obstetricia i Neonatologia (ICGON), Casa Maternitat, Barcelona, Spain; 5 Servei de Ginecologia y Obstetricia, Hospital de Sant Joan de Déu, Martorell, Spain; 6 Department of Epidemiology, Hospital de la Santa Creu i Sant Pau, Barcelona, Spain; 7 Program in Public Health and the Methodology of Biomedical Research, Universitat Autónoma de Barcelona (UAB), Cerdanyola del Vallès, Barcelona, Spain; 8 CIBER en Epidemiología y Salud Pública (CIBERESP), Barcelona, Spain

## Abstract

**Background:**

Studies on HPV infection in pregnant women and HPV transmission to the child have yielded inconsistent results.

**Methods:**

To estimate mother-to-child HPV transmission we carried out a prospective cohort study that included 66 HPV-positive and 77 HPV-negative pregnant women and their offspring attending a maternity hospital in Barcelona. To estimate HPV prevalence and genotype distribution in pregnancy we also carried out a related screening survey of cervical HPV-DNA detection among 828 pregnant women. Cervical cells from the mother were collected at pregnancy (mean of 31 weeks) and at the 6-week post-partum visit. Exfoliated cells from the mouth and external genitalia of the infants were collected around birth, at the 6-week post-partum visit, and around 3, 6, 12, and 24 months of age. All samples were tested for HPV using PCR. Associations between potential determinants of HPV infection in pregnant women and of HPV positivity in infants were also explored by logistic regression modelling.

**Results:**

Overall cervical HPV-DNA detection in pregnant women recruited in the HPV screening survey was 6.5% (54/828). Sexual behavior-related variables, previous histories of genital warts or sexually transmitted infections, and presence of cytological abnormalities were statistically significantly and positively associated with HPV DNA detection in pregnant women recruited in the cohort. At 418 infant visits and a mean follow-up time of 14 months, 19.7% of infants born to HPV-positive mothers and 16.9% of those born to HPV-negative mothers tested HPV positive at some point during infants' follow-up. The most frequently detected genotype both in infants and mothers was HPV-16, after excluding untyped HPV infections. We found a strong and statistically significant association between mother's and child's HPV status at the 6-week post-partum visit. Thus, children of mothers' who were HPV-positive at the post-partum visit were about 5 times more likely to test HPV-positive than children of corresponding HPV-negative mothers (p = 0.02).

**Conclusion:**

This study confirms that the risk of vertical transmission of HPV genotypes is relatively low. HPV persistence in infants is a rare event. These data also indicate that vertical transmission may not be the sole source of HPV infections in infants and provides partial evidence for horizontal mother-to-child HPV transmission.

## Background

Despite the overwhelming evidence for a sexual transmission of high-risk HPVs, other routes of transmission have been proposed. Several studies have explored whether HPVs can be vertically transmitted from mother to child by direct contact during labor, or horizontally through manipulation of the child with infected hands, bathing, towels and fomites (reviewed in [1,2]). Studies evaluating transmission of HPV from mother to infant and HPV persistence in newborns and children are conflicting and show a wide range of rates. Furthermore, a number of studies report HPV-DNA detection in infants born to HPV-DNA negative mothers. Thus, the mode of HPV acquisition in children, including vertical and horizontal transmission, remains a controversial issue [3].

To assess in a low-risk country the prevalence and determinants of HPV infection in pregnant women as well as perinatal transmission and persistence of HPV types, we carried out an HPV screening survey in pregnant women and a prospective study of HPV-positive and HPV-negative mothers and their infants.

## Methods

### Recruitment of subjects

The project was initiated in 1995 by carrying out a prospective cohort study of pregnant women attending a public maternity hospital for prenatal care in urban Barcelona, Spain. The hospital covers mostly general, non-selected population in its catchment area. Pregnant women were selected according to their potential risk of HPV exposure as based on their history of high-risk sexual behavior, genital warts, abnormal colposcopy, and HPV-related cervical lesions by cytology or histology. Emphasis was placed in recruiting high-risk women to procure a high yield of HPV-positive mothers and thus increase statistical power to properly study HPV transmission. A total of 115 consenting pregnant women were included and classified according to their risk of HPV exposure into high- (n = 73) and low-risk (n = 42) groups. At their first or second obstetric visit (mean pregnancy time 32.1 weeks, rank 30 to 34 weeks), consenting women were tested for cervical HPV-DNA status using a screening consensus primer PCR (see below). Since the yield of HPV-positive pregnant women was too low (4 of 42 women and 24 of 73 women from the low- and high-risk group, respectively) to adequately assess mother-to-child HPV transmission, we extended the study by carrying out an HPV screening survey among unselected pregnant women to identify and recruit additional HPV-positive subjects for the prospective cohort study.

### HPV screening survey among unselected pregnant women

Subjects for this HPV screening survey included 858 pregnant women consecutively attending the prenatal care clinic of the maternity hospital between 1997 and 2000. Consenting women were tested for cervical HPV-DNA status at their obstetric visit (mean pregnancy time 31 weeks, rank 29 to 33 weeks) using the same consensus primer PCR (see below) as that used in the initial cohort study. Women were included in the prospective cohort study only when a positive result was found (i.e. HPV-negative pregnant women from this HPV screening survey were not included in the prospective cohort study). Participation rates were not estimated and refusals were replaced by the next consenting women. This limitation should not bias our results as the purpose of the HPV screening survey was mainly the recruitment of additional HPV positive pregnant women to the prospective cohort study. Thirty women were excluded because the cervical samples collected were invalid for PCR testing leaving a total number of 828 women for the analysis of HPV prevalence among unselected pregnant women. Women from this survey that were not recruited into the prospective cohort study (those testing HPV-negative) did not receive the risk-factor questionnaire (see below).

### Prospective cohort study

Women in the final prospective cohort study included those from the initial cohort (n = 115) and all HPV-positive women from the HPV screening survey (n = 54) along with their offspring. At the enrollment visit, performed between 2 and 12 weeks after the HPV screening visit, all 169 women signed a written informed consent and were administered a full standardised questionnaire by personal interview that included socioeconomic, reproductive, sexual behavior, and risk factor variables. A standardised clinical form was also used to systematically collect all relevant clinical parameters during pregnancy, labor, and follow up visits. The questionnaires and clinical forms were only collected for the women participating in the prospective cohort study but not for the group of women that tested HPV-negative in the HPV screening survey.

### Cervical samples and follow-up

Cervical samples from each woman were collected using a dry swab (Eurotubo^® ^collection swab, Spain), at two time points: at the pregnancy visit (mean pregnancy times: 32.1 weeks for women in the initial cohort; and 31 weeks for women in the HPV screening survey), and at the 6-week postpartum visit. All samples were screened for cytological abnormalities.

Mouth and anogenital exfoliated cells from all infants were collected for HPV-DNA detection, using the same device as that used for mothers, at or around birth (between 0 and 6 days), at or around 6 weeks, and at or around 3, 6, 12, and 24 months of age. Genital samples from boys were obtained by gently rubbing the penis glans and the inner mucosal part of the prepuce. The penile shaft was not sampled. Genital samples from girls were obtained by gently rubbing throughout the mucosal part of the vulva. Mouth samples were obtained by firmly rubbing the dors of the tongue, mucosal part of the cheeks and the hard palate, using a single swab. Particular care was taken with sample collection procedures to prevent cross-contamination between subjects and different anatomical sites by using disposable equipment and changing bed lining and gloves for each woman. All the samples from recruited infants were mostly collected by the same pediatrician (JMP) throughout the study. Since the initial anal specimens did not yield sufficient DNA for adequate HPV testing, collection of anal samples was discontinued early in the follow-up and the few results obtained for this site were not considered in our analyses. All samples were sent to the laboratory, suspended in 1 mL of 0.1 M PBS (phosphate buffered saline), transferred to clean sterile eppendorf tubes and kept frozen at -20°C until processed.

At each visit a physical examination of the child that included also a clinical inspection of the oral cavity and the external anogenital area was performed.

### HPV-DNA detection and genotyping

DNA extraction was performed using 200 μL of cervical or oral cells suspensions and 400 μL from genital cells using the Qiamp Viral DNA kit (QIAGEN, Hilden, Germany) in accordance with the manufacturer's instructions.

Amplification was performed using 5 μL extracted DNA with the consensus primers MY09/MY11, which amplify a fragment from the L1 HPV gene [4]. PCR was performed in 50 μL of reaction mixture containing 2.2 mM MgCl2, 10 mM Tris HCl, 50 mM KCl, 2 μM of dNTPs, 1 μM of d-UTP-digoxigenin (DIG), 25 pmol of each primer and 1 U of Taq polimerase (Roche Diagnostics S.L.). PCR was carried out with the following cycling parameters: 95°C 1 min, 55°C 1 min, and 72°C 1 mim for 40 repeating cycles. PC04/GH20 primers were used to amplify a fragment of the Human β-globin gene as internal control of DNA integrity and absence of PCR inhibitors [5]. Generic HPV and type-specific biotinylated probes were immobilised on a strepavidin-coated microtiter plate. DIG-labeled PCR products were denatured, hybridised under stringent conditions, and detected using peroxidase-conjugated anti-digoxigenin antibody with colorimetric substrate. PCR products positive with the generic probe hybridization were also tested with HPV 6, 11, 16, 18, 31, 33 and 39 specific probes to asses type-specific positivity. Positive samples by the generic probe that failed to hybridize type-specific probes were classified as untyped and labeled HPV X.

All specimens were processed blindly to child or mother HPV-risk or HPV DNA initial status. Each PCR run included DNA extraction and HPV negative controls. Particular care was taken to prevent carry-over contamination by separating pre- and post-PCR areas.

### Statistical analyses

HPV prevalence was estimated among women at the pregnancy visit and at the 6-week postpartum visit. HPV prevalence estimates in pregnant women did not include those in the initial cohort study as these women were selected according to their risk of HPV exposure and would yield an overestimated HPV prevalence. Determinants of both HPV positivity in pregnant women and in their offspring were explored by multivariate unconditional logistic regression models to estimate prevalence odds ratios (ORs) and 95 percent confidence limits. ORs where adjusted by age in tertiles (<29, 29–33, >33). Even though these women were selected based on their HPV status and thus biased towards HPV positivity, this should not affect the validity of exploring potential risk factors for HPV infection in pregnant women.

HPV prevalence in infants and mother to child transmission rates were estimated at the perinatal period (0 to 6 days of life), at the 6-week postpartum visit and, since few HPV positive samples were detected after the 6-week visit, all childrens' HPV results from successive visits (3 months throughout 24 months) were combined in one single prevalence estimate. Overall and type-specific HPV transmission rates were also estimated at any point during follow-up. Estimates are given according to the HPV status of the mother at pregnancy and at the 6-week postpartum visit. HPV type-specific concordance among paired mother-child samples was estimated among the HPV-positive samples that could be genotyped. Paired samples with HPV X were not counted as concordant.

Two parameters were used to explore horizontal transmission: HPV positivity rates in infants born to HPV-negative mothers, and the correlation between HPV status in the mother and HPV status in the child in paired mother-child samples simultaneously collected at the 6-week visit.

All women participating in the cohort study signed a written informed consent. Protocols were approved by the local ethical committees of the Maternity Hospital, where the study was carried out, and the Ciutat Sanitaria i Universitaria de Bellvitge, where the coordinating center of the study was located.

## Results

### HPV detection among pregnant women

Of the initial 169 recruited pregnant women (115 from the initial cohort and 54 from the HPV screening survey), 82 (28 from the initial cohort and 54 from the HPV screening survey) were HPV positive at the pregnancy visit. The genotype distribution in these HPV-positive samples from pregnant women was in descending order of frequency: HPV 16 (32.9%), HPV X (28.0%), HPV 6/11 (25.6%), HPV 31 (11.0%), HPV 39 (9.8%), HPV 18 (7.3%), HPV 33 (3.7%). Multiple types were present in 14 women (17.1%). The most frequent combinations among these were co-infection of HPV 16 with other types (50.0%) and co-infection of HPV 31 with HPV 39 (35.7%). Cervical HPV prevalence in the unselected group of pregnant women from the HPV screening survey was 6.5% (54/828; 95% CI: 4.9%–8.4%).

### Baseline characteristics of subjects in the cohort

Of the initial 169 recruited women with a known HPV-DNA status, 26 mother-infant pairs (16 from the initial cohort and 10 from the HPV screening survey) were excluded from the prospective cohort study because no adequate sample from the child could be obtained for HPV testing neither at birth nor at any of the subsequent follow-up visits. Of the 26 excluded pairs, 16 (6 from the initial cohort and 10 from the HPV screening survey) were HPV positive. The distribution of HPV types in these 16 excluded HPV-positive women did not statistically differ from the 66 HPV-positive women finally included in the cohort (p = 0.23). Thus, the final prospective cohort study included 143 mother-infant pairs with a valid PCR result.

A total of 418 study visits were performed with a mean follow-up time of 14 months. All pregnancies ended on single births.

The characteristics of the 143 women and infants enrolled in the prospective cohort study along with the obstetric clinical details are detailed in Table 1.

**Table 1:** Baseline characteristics of the 143 women included in the prospective cohort study.

Characteristics	Number (n = 143)	%
**Age**		
≤ 25	22	15.4
26–30	45	31.5
31–35	52	36.4
≥ 36	24	16.8
**Marital status**		
Married	99	69.2
Cohabiting	37	25.9
Single	2	1.4
Separated	5	3.5
**Number of previous pregnancies**		
0	46	32.2
1	47	32.9
2	34	23.8
3	11	7.7
≥ 4	5	3.5
**Parity**		
0	65	45.5
1	53	37.1
2	22	15.4
3–4	3	2.1
**Weeks of pregnancy at delivery**		
≤ 37	13	9.1
38–39	80	55.9
≥ 40	50	35.0
**Type of delivery**		
Eutocic	86	60.1
Instrumental	38	26.6
Cesarean section	19	13.3
**Duration of delivery (hours)**		
< 1	13	9.1
1–5	75	52.4
6–10	47	32.9
>10	8	5.6
**Fetal stress during labor**		
Yes	11	8.0
No	127	92.0
Unknown	5	

As shown in Table 2 of the 143 pregnant women included in the cohort, 46.2% (66/143) tested positive for HPV-DNA during pregnancy. Excluding untyped infections, HPV 16 singly (25.8%) or in combination with other types (10.6%) was the most frequently detected type, followed by HPV 6/11 (15.2%), HPV 31 singly or with other types (9.1%), HPV 18 (6.1%), HPV 33 singly or with other types (3%), and HPV 39 (1.5%). The proportion of untyped infections (HPV X) was 28.8%.

**Table 2:** HPV results in mothers and infants included in the prospective cohort study.

	Number (n = 143)	%	(95% CI)
**Mothers' HPV-DNA status at pregnancy**			
Positive/Tested	66/143	46.2	(37.8–54.7)
Type distribution among HPV positive:			
HPV 6,11	10	15.2	
HPV 16	17	25.8	
HPV 18	4	6.1	
HPV 31	1	1.5	
HPV 33	1	1.5	
HPV 39	1	1.5	
HPV 16 + other types	7	10.6	
HPV 31 + other types	5	7.6	
HPV 33 + other types	1	1.5	
HPV X	19	28.8	
			
**Mothers' HPV-DNA prevalence at 6 weeks postpartum**			
Positive/Tested	25/118	21.2	(14.2–29.7)
Type distribution among HPV positive:			
HPV 6,11	4	16.0	
HPV 16	8	32.0	
HPV 18	2	8.0	
HPV 16 + other types	2	8.0	
HPV 18 + other types	1	4.0	
HPV 31 + other types	2	8.0	
HPV 39 + other types	1	4.0	
HPV X	5	20.0	
			
**Infants' HPV-DNA positivity (positive/tested)**			
Between 0 and 6 days	7/117	6.0	(2.4–11.9)
6 weeks after birth	14/110	12.7	(7.1–20.4)
Between 3 and 24 months	8/88	9.1	(4.0–17.1)
At any point (0 to 24 months)	26/143	18.2	(12.2–25.5)
Type distribution among HPV positive (at any point during follow-up):			
HPV X	10	38.5	
HPV 16	8	30.8	
HPV 6/11	6	23.1	
HPV 18	1	3.8	
HPV 31	1	3.8	
			
**Infants' HPV positivity by mother's HPV-DNA status (positive/tested)**			
Born to HPV negative mothers	13/77	16.9	(9.3–27.1)
Born to HPV positive mothers	13/66	19.7	(10.9–31.3)
			
**Origin of HPV positive samples in children**			
Oral	16	51.6	
Genital	15	48.4	

### Determinants of HPV infection in pregnant women in the cohort

The age-adjusted analyses to identify determinants of cervical HPV infection in pregnant women yielded the expected pattern of established risk factors for HPV infection (Table 3). No associations were found with marital status, tobacco smoking, parity, OC use and dietary variables (data not shown). The number of sexual partners before age 20, a history of previous STDs, and, to a lesser extent, an early age at first sexual intercourse, were the determinants most strongly related to HPV-DNA detection during pregnancy.

**Table 3:** Selected determinants of HPV infection in pregnant women recruited in the prospective cohort study.

	HPV Positive/tested (n = 66/143)	HPV-positive (%)	OR	(95% CI)
**Age**				
≤ 20	3/4	75.0	1.0	Reference
21–25	10/18	55.6	0.42	(0.04–4.82)
26–30	17/45	37.8	0.20	(0.02–2.11)
31–35	21/52	40.4	0.23	(0.02–2.32)
≥ 36	15/24	62.5	0.56	(0.05–6.19)
*Chi-square p-value*	*0.16*			
				
**Marital Status**				
Married	41/99	41.4	1.0	Reference
Cohabiting	20/37	54.1	1.78	(0.81–3.90)
Single	1/2	50.0	1.62	(0.10–27.30)
Separated	4/5	80.0	4.09	(0.42–40.26)
*Chi-square p-value*	*0.25*			
				
**Age at first intercourse**				
≤ 17	27/42	64.3	1.0	Reference
18–19 yrs	15/35	42.9	**0.38**	**(0.15–1.00)**
≥ 20 yrs	13/37	35.1	**0.29**	**(0.11–0.76)**
Unknown	11/29			
*Chi-square p-value*	***0.03***			
				
**No. of sexual partners before 20 years of age**				
0	5/24	20.8	1.0	Reference
1	29/58	50.0	**3.45**	**(1.12–10.67)**
≥ 2	21/32	65.6	**7.23**	**(2.05–25.50)**
Unknown	11/29			
*Chi-square p-value*	***0.004***			
				
**Lifetime no. of sexual partners**				
1	17/44	38.6	1.0	Reference
2–4	17/39	43.6	1.22	(0.50–2.95)
≥ 5	21/31	67.7	**3.17**	**(1.19–8.41)**
Unknown	11/29			
*Chi-square p-value*	***0.04***			
				
**Previous history of genital warts**				
No	28/70	40.0	1.0	Reference
Yes	23/39	59.0	**2.15**	**(0.96–4.82)**
Unknown	15/34			
*Chi-square p-value*	***0.06***			
				
**Previous history of STD**				
No	37/92	40.2	1.0	Reference
Yes	14/17	82.4	**6.82**	**(1.82–25.62)**
Unknown	15/34			
*Chi-square p-value*	***0.001***			
				
**Cytology results**				
Normal	49/120	40.8	1.0	Reference
ASCUS	1/1	100	1.0	
≥ LSIL	12/17	70.6	**3.15**	**(1.03–9.61)**
Unknown	4/5			
*Chi-square p-value*	***0.02***			

Models adjusted by age (≤ 28, 29–33, ≥ 34 years)

As expected we found a strong association between HPV positivity and presence of cervical lesions (LSIL or worse). All HSIL cases tested HPV positive being HPVs 16, 18, and HPV X the most frequently detected types among women with HSIL.

### HPV positivity in infants

At 418 infant visits and a mean follow-up time of 14 months, overall prevalence of HPV in infants at any visit was 18.2% (Table 2). The most frequently detected types in infants after excluding untyped infections were HPV 16, followed by HPV 6/11 and HPV 18 and 31. HPV positivity in infants was not related to the site from which the samples were collected: 51.6% versus 48.4% of the oral and genital specimens were positive for HPV-DNA, respectively. HPV status concordance between oral and genital samples was 93%. Of the 26 infants that tested HPV positive at any point during follow-up, a valid PCR result from both the oral and the genital sites was obtained in 26 pairs. In 24 of these oral-genital pairs the HPV status was discordant, in one pair the detected types were concordant for HPV 11 and in the other pair the types were not concordant.

### Determinants of HPV infection in children

Table 4 summarizes the results of the analyses exploring putative risk factors for HPV infection in children at any time during follow-up. None of the clinical, obstetric and sexual behavior-related characteristics of the mother was associated with HPV detection in the children. The only determinants associated with HPV-DNA detection in the offspring were the mother's HPV status at the postpartum visit and, inversely, mother's past use of hormonal contraception. Thus, at the 6-week post-partum visit, children of mothers who were HPV-positive at the post-partum visit were about 5 times more likely to test HPV-positive than children of corresponding HPV-negative mothers (27.3% vs. 7.2%, respectively; age-adjusted OR = 4.8; 95% CI, 1.4–16.9; p = 0.02). Mothers testing HPV positive both at the pregnancy visit and at the post-partum visit, were twice more likely than mothers testing negative at both visits to have HPV-positive children at any time during follow-up (29.2% versus 15.4%, respectively: age-adjusted OR = 2.3, 95% CI, 0.8–7.1). However, the increased risk didn't reach statistical significance.

**Table 4:** Selected determinants of HPV DNA detection in children at any time during follow up.

Mother's and pregnancy characteristics	HPV Positive/Tested	HPV-positive children (%)	OR	(95% CI)
**Total**	26/143	18.2%		
**Age at delivery**				
<26	5/22	22.7	1.0	Reference
26–30	9/45	20.0	0.85	(0.25–2.93)
31–35	8/52	15.4	0.62	(0.18–2.16)
>35	4/24	16.7	0.68	(0.16–2.94)
*Chi-square p-value*	0.87			
**Weeks of pregnancy at delivery**				
<38	3/13	23.1	1.0	Reference
38–39	15/80	18.8	0.77	(0.19–3.14)
>39	8/50	16.0	0.64	(0.14–2.84)
*Chi-square p-value*	0.82			
**Type of delivery**				
Eutocic	12/86	14.0	1.0	Reference
Instrumental	10/38	26.3	2.20	(0.85–5.68)
Cesarean section	4/19	21.1	1.60	(0.45–5.75)
*Chi-square p-value*	0.24			
**Duration of delivery (hours)**				
<1	3/13	23.1	1.0	Reference
1–5	13/75	17.3	0.71	(0.17–2.97)
6–10	7/47	14.9	0.60	(0.13–2.83)
>10	3/8	37.5	2.10	(0.28–15.57)
*Chi-square p-value*	0.46			
**Marital status**				
Married or Cohabiting	25/136	18.4	1.0	Reference
Single or Separated	1/7	14.3	0.78	(0.09–7.10)
*Chi-square p-value*	0.78			
**Age at first intercourse**				
≤ 17	9/42	21.4	1.0	Reference
18–19	7/35	20.0	0.89	(0.28–2.79)
≥ 20	6/37	16.2	0.67	(0.21–2.20)
Unknown	4/29			
*Chi-square p-value*	0.84			
**Number of previous pregnancies**				
0	9/46	19.6	1.0	Reference
1	8/47	17.0	0.89	(0.31–2.61)
2	5/34	14.7	0.79	(0.22–2.79)
≥ 3	4/16	25.0	1.69	(0.35–8.29)
*Chi-square p-value*	0.83			
**Lifetime no. of sexual partners**				
1	9/44	20.5	1.0	Reference
2–4	8/39	20.5	1.01	(0.35–2.94)
≥ 5	5/31	16.1	0.76	(0.23–2.57)
Unknown	4/29			
*Chi-square p-value*	0.87			
**Previous history of STD**				
No	15/92	16.3	1.0	Reference
Yes	5/17	29.4	2.19	(0.67–7.16)
Unknown	6/34			
*Chi-square p-value*	0.20			
**Hormonal contraceptive use**				
Never	10/26	38.5	1.0	Reference
Ever	12/88	13.6	**0.24**	**(0.09–0.67)**
Unknown	4/29			
*Chi-square p-value*	**0.005**			
**Tobacco smoking status**				
Never smoker	7/33	21.2	1.0	Reference
Ex-smoker	5/20	25.0	1.21	(0.32–4.52)
Current smoker	10/66	15.2	0.65	(0.22–1.96)
Unknown	4/24			
*Chi-square p-value*	0.55			
**Cytology results at pregnancy**				
Normal-ASCUS	21/121	17.4	1.0	Reference
LSIL-HSIL	3/17	17.6	1.04	(0.27–3.98)
Unknown	2/5			
*Chi-square p-value*	0.98			
**Mother's HPV status at pregnancy**				
Negative	13/77	16.9	1.0	Reference
Positive	13/66	19.7	1.25	(0.53–2.94)
*Chi-square p-value*	0.66			
**Mother's HPV status at post-partum visit**				
Negative	12/93	12.9	1.0	Reference
Positive	8/25	32.0	**3.22**	**(1.14–9.10)**
*Unknown*	6/25			
*Chi-square p-value*	**0.02**			
**Mother's HPV status at pregnancy and/or post-partum visit**				
Negative at both visits	10/65	15.4	1.0	Reference
Positive at either visit	7/43	16.3	1.09	(0.38–3.14)
Positive at both visits	7/24	29.2	2.33	(0.76–7.10)
Unknown	2/11			
*Chi-square p-value*	0.30			

Models were adjusted by age group (≤ 28, 29–33, ≥ 34). No association was found with fetal stress at delivery, parity, duration and intensity of smoking, child's sex, no. of sex partners before or after 20 years of age or history of genital warts.

### Mother to child HPV transmission

As shown in Table 5, 19.7% of the 66 infants born to HPV-positive mothers and 16.9% of the 77 infants born to HPV-negative mothers tested HPV-DNA positive at some point during follow-up (p = 0.7). As also shown in Table 4, we found a statistically significant association between HPV status in mothers' and children's samples collected at the 6-week visit: 27.3% versus 7.2%, respectively (p = 0.02). We also found an association between mother's HPV status at the post-partum visit and infant's HPV positivity at any time during follow-up (p = 0.03). There was no association between mother's HPV status at pregnancy and child's HPV status at any of the follow-up visits. Table 5 also shows that the type-specific concordance in paired mother-child samples was low at any of the visits. The overall distribution of HPV types among mothers at pregnancy was not statistically significantly different from that among infants at any time (p = 0.80)

**Table 5:** Percentage of HPV positive infants at different points during follow-up according to mother's HPV status and specific genotypes detected in mother/child HPV positive pairs.

	Infants' HPV positivity by follow-up period			
	
Mothers' HPV status	at 0 to 6 days	at 6 weeks	at 3 to 24 months	Any time (0 to 24 months)
	
	% (HPV+/tested)	% (HPV+/tested)	% (HPV+/tested)	% (HPV+/tested)
	
At pregnancy				
HPV negative	3.1% (2/64)	11.9% (7/59)	7.4% (4/54)	16.9% (13/77)
HPV positive	9.4% (5/53)	13.7% (7/51)	11.8% (4/34)	19.7% (13/66)
Mother/child HPV positive pairs with concordant genotypes [types in mother/child pairs]	1 of 5 [16,6,11/6,11]	2 of 7 [16/16; 18/18]	2 of 4 [16/16; 18/18]	4 of 13 [16,6,11/6,11; 16/16; 16/16; 18/18]

Mother/child HPV positive pairs with discordant genotypes [types in mother/child pairs]	4 of 5 [18/6,11; X/X; X/X; X/31]	5 of 7 [18/6,11; 18/6,11; X/X; X/X; 16/X]	2 of 4 [6,11/16; X/X]	9 of 13 [18/6,11; 18/6,11; 6,11/16; X/31; X/X; X/X; X/X; X/X; X/X]

Fisher's exact test*	*0.2*	*0.8*	*0.7*	*0.7*

**At 6 weeks postpartum**				
**HPV negative**	2.7% (2/74)	7.2% (6/83)	6.2% (4/65)	12.9% (12/93)
**HPV positive**	11.1% (2/18)	27.3% (6/22)	21.4% (3/14)	32.0% (8/25)

Mother/child HPV positive pairs with concordant genotypes [types in mother/child pairs]	0 of 2	2 of 6 [18,6,11/6,11; 18/18]	2 of 3 [18/18; 16,6,11/16]	3 of 8 [18,6,11/6,11; 18/18; 16,6,11/16]

Mother/child HPV positive pairs with discordant genotypes [types in mother/child pairs]	2 of 2 [16/X; 18/6,11]	4 of 6 [18/6,11; X/X; 16,6,11/X; X/6,11]	1 of 3 [6,11/16]	5 of 8 [18/6,11; 6,11/16; X/X; 16/X; X/6,11]

Fisher's exact test*	*0.2*	***0.02***	*0.1*	***0.03***

* For the association between HPV status in mother and HPV status in child

### HPV persistence in mothers and infants

A total of 118 women had valid PCR results both at pregnancy and at the post-partum visit. Among women HPV-positive at pregnancy HPV status persistence up to the post-partum visit was 46.2% (24/52). New infections at the post-partum visit among HPV-negative women at pregnancy occurred in only one woman (1.5%, 1/66). Thus, there was a strong association between HPV status at pregnancy and HPV status in the mother at the post-partum visit (p < 0.0001). Type-specific concordance among HPV-positive women between pregnancy and post-partum visit was 100% (17/17 after excluding 7 women with HPV X at either visit). The overall type-specific distribution of HPV-positive mothers at pregnancy was not statistically significantly different from that at the post-partum visit (p = 0.83). Of the 19 mothers that tested HPV X at pregnancy only 2 were HPV X persistent at the post-partum visit, 9 became HPV negative, 3 became HPV-16 positive, and in the remaining 5, no PCR result was available at the post-partum visit.

Of the 26 children that tested HPV positive at some point during follow-up, 18 had at least two consecutive samples to assess HPV persistence. Three (16.7%) had two consecutive HPV-positive samples and in two of them (11.1%) there was genotype-specific persistence. In the remaining 15 infants (83.3%) their first HPV-positive sample was followed by a subsequent HPV-negative sample. There was no statistical evidence that the distribution of HPV types detected from the infants changed over time (data not shown). At the end of the study none of the infants developed oral, anogenital or cutaneous, macroscopically identifiable HPV-related lesions.

## Discussion and Conclsusion

Our data from both the HPV screening survey of unselected pregnant women and the prospective cohort study provide for the first time estimates for HPV prevalence, type-specific distribution, mother-to-child transmission rates as well as HPV persistence in pregnant women and in infants in Spain.

Consistent with previous reports (reviewed in [2,3,6-8]), our data confirm that the risk of transmission of any HPV type from infected mothers to the newborn is shown to be relatively low (9.4%), even lower (2.0%) if type-specific transmission is considered.

Although HPV-DNA detection rates in samples of newborns and infants vary widely in the literature, well conducted prospective studies suggest that the risk of perinatal transmission, although existent, is relatively low. Several studies have tested infants for HPV-DNA or antibodies ([9-13] and reviews [2,3,6-8]). Detection rates in the first 1 or 2 days of life range between 4% and 72% among infants born to women with genital HPV detected during pregnancy, and between 0.6% and 20% among infants delivered by women with no detectable HPV during pregnancy. Rates of detection at 6 weeks vary also widely and they are not always significantly different for infants born to HPV positive or negative mothers.

In a carefully conducted large study [13] the HPV prevalence among infants born to HPV-positive women was 4% and among infants born to HPV-negative women was 8%. Consistent with our high rates of HPV X detection, this study found that all positive results in the infants were positive for unclassified HPV types and all of them were preceded or followed by HPV negative specimens. This report and our data clearly show that the few HPV infections detected in infants probably represent low-level genital or non-genital HPVs or may represent horizontal transmission. Taken together the evidence from this and other prospective studies [9,10] strongly suggests that the risk of perinatal transmission of HPVs although existent is relatively low.

A consistent finding from our cohorts of HPV positive and negative pregnant women and their offspring is the evidence for horizontal transmission. First, we found that up to 16.9% of children born to HPV-negative mothers had HPV infections in the first 24 months of life. This percentage is only slightly lower, and not statistically significantly different, than that observed in infants born to HPV-positive mothers (19.7%). Secondly, we found an association between HPV status in the mother at the 6-week postpartum visit and the HPV status in children at the same visit or thereafter. Thus, at the 6-week post-partum visit, children of mothers' who were HPV-positive at the post-partum visit were about 5 times more likely to test HPV-positive than children of corresponding HPV-negative mothers (27.3% vs. 7.2%; age-adjusted OR = 4.8; 95% CI, 1.36–16.88; p = 0.02). In contrast, no association was found between mothers' HPV status at pregnancy and children's HPV status at any of the visits combined. Thus, all together, the data in Table 5 indicate that the HPV detected at the post-partum visit in the mother is a stronger determinant of HPV infection in the child than the HPV detected during pregnancy, suggesting that horizontal mother-to-child transmission may play a more important role than vertical transmission in determining HPV DNA detection in children. Mothers themselves, relatives, caregivers and fomites harbour HPVs that can be horizontally transmitted to the child, in particular in the first weeks of life when there is a close caring physical contact relationship with the infant. Indeed, other study designs are needed to properly distinguish vertical from horizontal transmission. These studies should include accurate and repeated HPV detection and genotyping of multiple sites from parents, siblings and care givers as well as assays to distinguish between markers of inert HPV DNA detection and markers of active HPV infection.

In assessing HPV positivity in children born to HPV-negative mothers we can not rule out that these mothers were false HPV negatives at pregnancy. We need to take into account that 52% of the HPV-negative mothers came from the high-risk group of women included in the initial cohort study. Thus, increased HPV exposure may increase risk of false negative results which might somehow partially explain HPV transmission among HPV-negative mothers.

Our results do not support a high prevalence of HPV during pregnancy: 6.5% of the unselected group of women was positive for HPV-DNA by consensus PCR. This relatively low HPV prevalence in pregnant women correlates with the low prevalence of HPV infection in the female general population (between 1.3% and 2.4%) [14,15] and the low incidence rate of cervical cancer in Spain [15]. Still, our HPV prevalence estimate among pregnant women is between 3 and 5 times higher than that observed in the female general population, confirming the findings from other studies showing that pregnant women do have a higher HPV-DNA detection rate than un-pregnant women [6,16-18]. It has been argued that immunological or hormonal changes could modulate the rate of HPV positivity and clearance during pregnancy [18,19]. While some authors report evidence that pregnancy decreases clearance of high-risk HPV types in the first two trimesters of pregnancy [16,18,19], others question these findings [20,21].

As expected, and consistent with the sexual mode of transmission of HPV infections, we found that an early age at first sexual intercourse, a high number of sexual partners, particularly before the age of 20, a previous history of genital warts or STDs as well as a current squamous intraepithelial lesion in the cervix as diagnosed by cytology, were the strongest determinants of HPV positivity in pregnant women (Table 3). These expected associations provide further internal validity to our complex study.

As shown in Table 4 none of the obstetric variables collected in the study, including cesarean section delivery, were associated with HPV positivity in children at any time during follow-up. The effect of cesarean section on HPV transmission among HPV-positive pregnant women could not be assessed due to the low number of HPV-positive children born by cesarean section. Concerning reproductive variables, only ever use of hormonal contraception was associated with a reduced risk of HPV in the child. We do not have any biologically plausible explanation for this inverse association. Since such a relationship has never before been reported one should be cautious in its interpretation. Concerning HPV in the mother the only correlate found for HPV positivity in the child at any point during follow-up was the mother's HPV positivity at the post-partum visit. Again, this finding suggests that HPV at the post-partum visit may be more determinant for child's HPV infections than HPV at pregnancy. It is worth noting that mothers that had HPV status persistence from pregnancy to the post-partum visit had a higher percentage of HPV-positive children (29.2%) as compared to mothers negative at the two visits (15.4%) or HPV positive in only one of the two visits (16.3%). However, the association did not reach statistical significance (Table 4).

In interpreting these results it should be considered that our reported HPV type-specific distribution is probably biased towards an overestimated detection of HPV X, as the samples that tested positive with the generic probe were tested with only seven type-specific probes (HPVs 6,11,16,18,31,33, and 39). Thus the high percentage of samples classified as HPV X could be true rare genital HPV types, cutaneous types (unlikely because of the poor efficiency of the primer system in detecting cutaneous types) or still untyped HPVs. Furthermore the MY09/MY11 primer system has been improved by the PGMY primer system in terms of higher reproducibility of primer synthesis as well as detection of mucosal HPVs. Unfortunately, very few samples remained available for re-testing with the newer PGMY system. This limitation may also have resulted in an underestimation of the true underlying type-specific concordance.

In conclusion, our study, conducted in a population at low risk for HPV and cervical cancer, confirms that high-risk HPV genotypes can be vertically transmitted to the child, although the risk of vertical transmission is relatively low. In this study, if we exclude untyped (HPV X) infections, HPV 16 has been found to be the most frequent type detected both in mothers and infants. Infants of women who tested HPV positive at six weeks after delivery are nearly five times more likely to test HPV positive than infants of HPV-negative mothers. HPV persistence in infants is a rare event. Given the substantial HPV positivity observed in children born to HPV-negative mothers, these data suggest that vertical transmission may not be the sole source of HPV infections in children and that horizontal mother-to-child transmission may play also a role. It remains to be seen whether this alternative mode of HPV transmission and acquisition may have an impact in several areas, including vaccination strategies, epidemiological studies, and the clinical management of children with HPV-associated diseases.

## Competing interests

XC: Research Grants (GlaxoSmithKline, Merck Sharp & Dohme, Sanofi Pasteur MSD); Speakers Bureau (GlaxoSmithKline, Sanofi Pasteur MSD); Steering Committee (GlaxoSmithKline, Sanofi Pasteur MSD). GA: Travel grants (GlaxoSmithKline, Sanofi Pasteur MSD,). SdS: Research Grants (Merck & Co. Inc., Sanofi Pasteur MSD). FXB: Advisory Board (GlaxoSmithKline, Merck Sharp & Dohme, Sanofi Pasteur MSD); Speakers Bureau (GlaxoSmithKline); Research Grants (Merck Sharp & Dohme, Sanofi Pasteur MSD, Qiagen).

## Authors' contributions

XC, TD, FXB, SdS were the principal epidemiological investigators in the various phases of this long study, conceived the study, wrote the protocols, assured funding, identified clinical investigators and study personnel, supervised statistical analyses and performance of laboratory assays, and wrote the manuscript. MPC, TD wrote the HPV assays protocols, performed the HPV DNA detection and genotyping of the samples validating the techniques with other referent HPV laboratories, created the HPV data base, wrote part of the methods section and made substantial comments to the manuscript. AG, RR, JMP were the clinical investigators (two ObGyn and one pediatrician), trained and supervised study clinical staff (nurses, gynecologists and pediatricians) and sample collection throughout the study, and made substantial contributions to the manuscript. MJQ, JM, GA coordinated the field work in terms of study implementation and data collection, designed the study data collection forms, created clinical and laboratory databases, implemented quality assurance procedures, made the statistical analyses, produced the working and final tables and made substantial comments to the manuscript.

## Funding

The work was partially supported by Spanish public grants from the Instituto de Salud Carlos III (grants FIS PI93/0335, FIS PI98/0699, FIS PI030240, FIS PI061246, RCESP C03/09, RTICESP C03/10, RTIC RD06/0020/0095 and CIBERESP), from the Agència de Gestió d'Ajuts Universitaris i de Recerca (AGAUR 2005SGR 00695), and from the Marató de TV3 Foundation (051530), who had no role in the data collection, analysis or interpretation of the results.

## Pre-publication history

The pre-publication history for this paper can be accessed here:

http://www.biomedcentral.com/1471-2334/9/74/prepub
